# Biodegradability of woody film produced by solvent volatilisation of Japanese Beech solution

**DOI:** 10.1038/s41598-019-57228-7

**Published:** 2020-01-16

**Authors:** Yuri Nishiwaki-Akine, Sui Kanazawa, Norihisa Matsuura, Ryoko Yamamoto-Ikemoto

**Affiliations:** 1 Career Design Laboratory for Gender Equality, Kanazawa University, Kakuma-machi, Kanazawa 920-1192 Japan; 2 Division of Environmental Design, Graduate School of Natural Science and Technology, Kanazawa University, Kakuma-machi, Kanazawa 920-1192 Japan; 3 Faculty of Geosciences and Civil Engineering, Institute of Science and Engineering, Kanazawa University, Kakuma-machi, Kanazawa 920-1192 Japan

**Keywords:** Microbiology, Environmental sciences, Chemistry, Materials science

## Abstract

To address the problem of marine pollution from discarded plastics, we developed a highly biodegradable woody film, with almost the same components as wood, from the formic acid solution of ball-milled wood. We found that the woody film was not easily degraded by cultured solution of hand bacteria (phylum *Proteobacteria* was dominant). However, the film was easily biodegraded when in cultured solution of soil (*Firmicutes*, especially class *Bacilli*, was dominant) for 4 weeks at 37 °C, or when buried in the soil itself, both under aerobic conditions (*Acidobacteria* and *Proteobacteria* were dominant) for 40 days at room temperature and under anaerobic conditions (*Firmicutes*, especially family *Ruminococcaceae*, was dominant) for 5 weeks at 37 °C. Moreover, when film was buried in the soil, more carbon dioxide was generated than from soil alone. Therefore, the film was not only brittle but formed of decomposable organic matter. We showed that the film does not decompose at the time of use when touched by the hand, but it decomposes easily when buried in the soil after use. We suggest that this biodegradable woody film can be used as a sustainable raw material in the future.

## Introduction

In recent years, plastics dumped as garbage after use have often been released into the sea, leading to frequent ingestion of microplastics by marine organisms. Therefore, the low biodegradability of plastics has become a major social problem. Development of materials with high biodegradability is an important approach to help solve this problem. As a material with high biodegradability, there has been considerable research on biodegradable polymers^1^, and attempts are also being made to use wood powder^2^ and wood itself^3^ as a biodegradable plastic substitute.

In our previous research on sustainable biomass-derived materials, we found that transparent films can be obtained by solvent volatilisation from the solution of ball-milled wood dissolved in formic acid^4^. This woody film contains almost the same ingredients as wood and can be bended and folded without breaking^5^. We observed through scanning electron microscopy that the surface of the film was smooth. The film had a relatively high Young’s modulus and tensile strength and therefore we believe that it can be used instead of plastics. Thermal analysis also showed that the woody film can be used up to 180 °C without softening. Furthermore, the film was highly biodegradable, and thus it assimilated with soil approximately 6 weeks after burial^5^. This is a rapid biodegradation compared with well-known biodegradable polymers such as polylactic acid.

The wood itself is known to be readily biodegraded by fungi^6^ and bacteria^7^. The main components of the wood are cellulose, hemicellulose and lignin. Examples of fungi that biodegrade wood are white rot fungi (which can decompose cellulose and lignin)^8^ and brown rot fungi (which can decompose cellulose). Examples of bacteria that biodegrade wood are as follows: *Actinobacteria* (aerobic) and *Firmicutes* (anaerobic) are known for cellulose decomposition; *Acidobacteria*, *Proteobacteria* and *Actinobacteria* are known for hemicellulose decomposition; and *Actinobacteria* and *Proteobacteria* are known for lignin decomposition^9^. Cellulolytic bacteria which decompose cellulose include *Cellulomonas* (*Actinobacteria*) and *Streptomyces* (*Actinobacteria*)^10,11^, which are Gram positive bacteria. In addition, some types of *Ruminococcus* are known to be able to decompose cellulose^12^. Examples of bacteria that decompose lignin include *Pseudomonas* (*Proteobacteria*), *Xanthomonas* (*Proteobacteria*), *Bacillus* (*Firmicutes*), *Streptomyces* (*Actinobacteria*) and *Nocardia* (*Actinobacteria*)^13–16^.

Woody transparent film also contains cellulose, hemicellulose and lignin, therefore, it is necessary to determine whether the bacteria that can biodegrade the film are the same as the cellulose, hemicellulose and lignin-degrading bacteria.

We were concerned that highly biodegradable materials would be easily degraded by bacteria during use and therefore they might be less durable than the equivalent plastic product. Therefore, we examined the degradability of the woody film by bacteria existing on our hand to confirm whether they could be degraded by bacteria during daily use. Furthermore, we studied the biodegradability of the film under various conditions of burial in soil by the bacteria causing biodegradation. If the woody film can be easily decomposed by simply burying it in the soil after use, we can show that the film is a highly biodegradable material that does not cause a negative environmental impact.

## Results and Discussion

### Immersion of the woody film in soil and hand cultures

Soil bacteria and natural hand bacteria were cultured in a liquid medium, and the degree of degradation of the woody film in cultures was observed. We analysed the microbial community of the cultured bacteria: the soil cultures mostly included the *Firmicutes* (*Bacilli*) and the hand cultures mostly included the *Proteobacteria* (*Gammaproteobacteria*) (Table 1). Within the *Gammaproteobacteria* in the hand bacteria, there were many *Enterobacteriales* (*Enterobacteriaceae*) and *Pseudomonadales* (*Pseudomonadaceae*) (Supplementary Table S1). In the hand culture, there were other bacteria such as *Clostridia* and *Bacilli* (*Firmicutes*). However, there was a difference between the *Bacilli* found in the hand culture and those found in the soil culture. The soil culture included mainly the *Bacillaceae* and *Staphylococcaceae* families, whereas the hand culture included the *Planococcaceae* and *Paenibacillaceae* families (Supplementary Table S1).

**Table 1:** Main constituents of soil cultures and hand cultures (class level).

	relative abundance	
	hand	soil
*k__Bacteria*		
*p__Proteobacteria;c__Gammaproteobacteria*	56.6%	0.02%
*p__Proteobacteria;c__Betaproteobacteria*	0.1%	
*p__Proteobacteria;c__Alphaproteobacteria*	0.003%	
*p__Proteobacteria;c__Deltaproteobacteria*		0.003%
*p__Proteobacteria;c__Epsilonproteobacteria*		0.003%
*p__Firmicutes;c__Bacilli*	26.2%	98.6%
*p__Firmicutes;c__Clostridia*	17.1%	0.03%
*p__Bacteroidetes;c__Bacteroidia*	0.01%	0.01%
*p__Planctomycetes;c__[Brocadiae]*	0.01%	
*p__Synergistetes;c__Synergistia*	0.003%	
*p__Actinobacteria;c__Actinobacteria*		1.3%
Total	100.0%	100.0%

**Figure 1:**
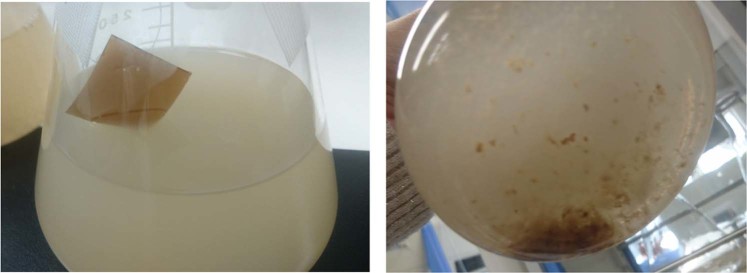
Woody film soaked in hand culture solution (left) and soil culture solution (right) (after 4 weeks).

We observed the degree of disintegration of the woody film in the culture. In the soil culture, the film collapsed and the original shape was not retained after 37 °C for 4 weeks; however, the film did not change in appearance, even if it was placed in hand culture under the same conditions (Fig. 1).

The original soil was collected from the same place (Kanazawa University) as that presented in Supplementary Tables S2 and S3 although the sampling date was different. In the data presented in Supplementary Tables S2 and S3, *Acidobacteria* and *Proteobacteria* were dominant in the original soil (Soil day 0). Therefore we presume that there were more *Acidobacteria* and *Proteobacteria* than *Firmicutes* (*Bacilli*) in the original soil. We consider that this increase in class *Bacilli* (family *Bacillaceae*) after culture was simply because the culture medium was suitable for *Bacilli* growth. However, the biodegradation of the woody film in soil culture showed that *Bacilli* also contributed to biodegradation.

Since *Bacillus subtilis* are known to produce cellulase^17^ and *Bacillus* that can degrade lignin are also known^15,16^, it is not surprising if bacteria from family *Bacillaceae* biodegraded woody film although we could not identify the genus and species in the *Bacillaceae* family in this study.

In addition, there are reports that many *Proprionibacteria* (*Actinobacteria*), *Streptococcaceae* (*Firmicutes*) and *Staphylococcaceae* (*Firmicutes*) exist in hand surface bacteria^18^. The most common bacterial phylum in human skin is the *Actinobacteria*, the second most common is *Firmicutes*, and the third most common is *Proteobacteria*^19,20^. However, this varies depending on the site, and there are more *Actinobacteria* on the face, abdomen and back, whereas *Proteobacteria* occurs more on hands and forearms^21^. The finding that *Proteobacteria* was the most common bacteria on the hand skin in this study was therefore considered accurate.

In this experiment, (even if there is a possibility that the ratio of microorganisms had changed by culture) the woody film was not biodegraded by cultured hand bacteria. Therefore, we predicted that the woody film would not be biodegraded in normal use (same as wood is not biodegraded in normal use). In fact, the woody film retained its original form even though it was touched once a day by hands for 4 weeks at room temperature in another test.

From the above results, we conclude that there are some bacteria that can degrade the woody film and some bacteria that cannot, and bacteria present in the soil can degrade the film.

### Film burial in soil

Based on the above results, the next burial tests were conducted to find out which bacteria in the soil contributed most to film degradation.

**Figure 2:**
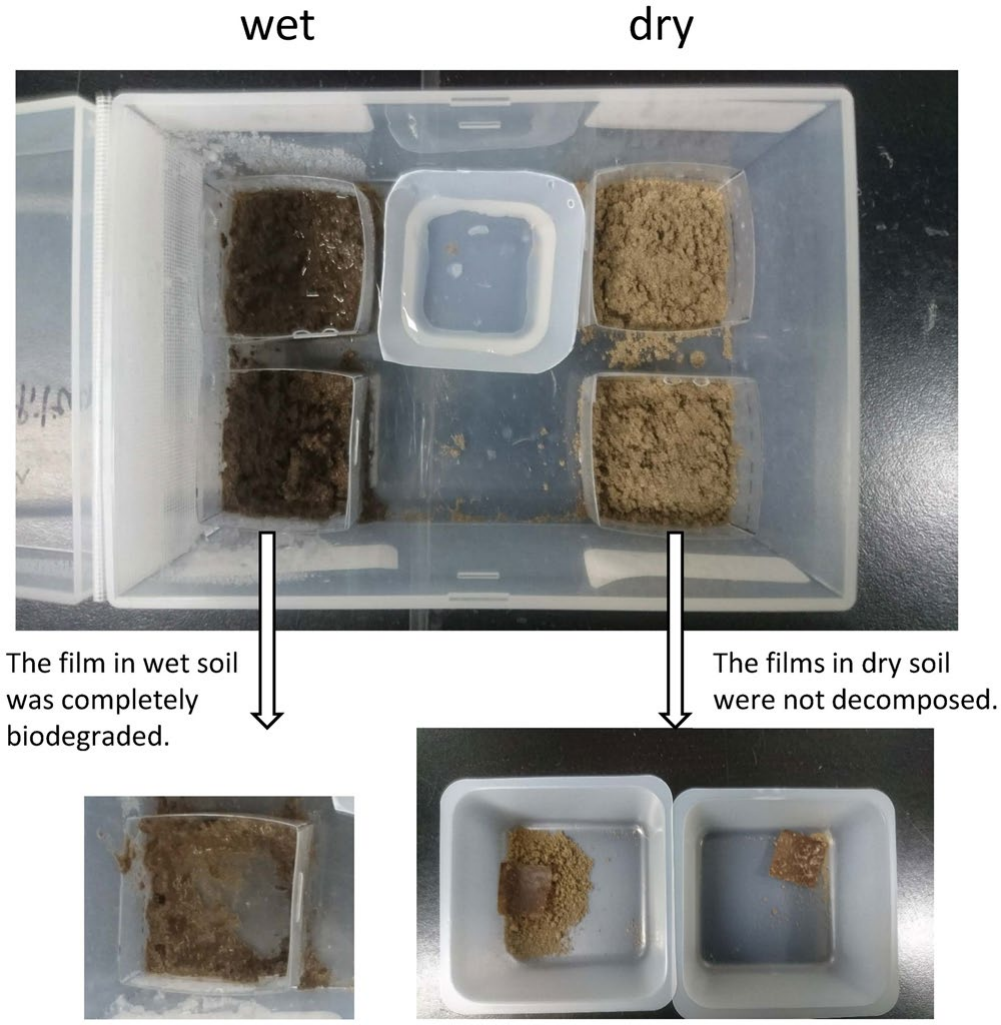
Samples after 40 days of film burial in the soil.

The first burial test was a simple film sandwich test for 40 days at room temperature. The woody film that was in the wet soil that was occasionally sprayed with water and not allowed to dry was completely biodegraded at a room temperature after 40 days. In contrast, the films that were in the dry soil (two samples) were not decomposed after 40 days (approximately 70% of the film remained; Fig. 2, Table 2). Therefore, we concluded that moisture promotes biodegradation of the woody film. However, a part of the film (about 30%) was biodegraded even under dry conditions after 40 days and this is faster biodegradation than that of general plastics. For example, polyethylene, general plastic, did not lose weight at all when buried in soil for 6 weeks^5^. In addition, in the previous report^5^, the woody film buried in soil at room temperature was also almost completely biodegraded after 6 weeks, although the conditions were slightly different. Thus, we confirmed that the biodegradability of the woody film in soil was replicable.

**Table 2:** Film remaining in wet and dry soil.

	day 0	40 days	
	mg	mg	%
film in wet soil	53.5	0	0%
film in dry soil 1	51.8	34.3	66%
film in dry soil 2	80.0	59.5	74%

**Figure 3:**
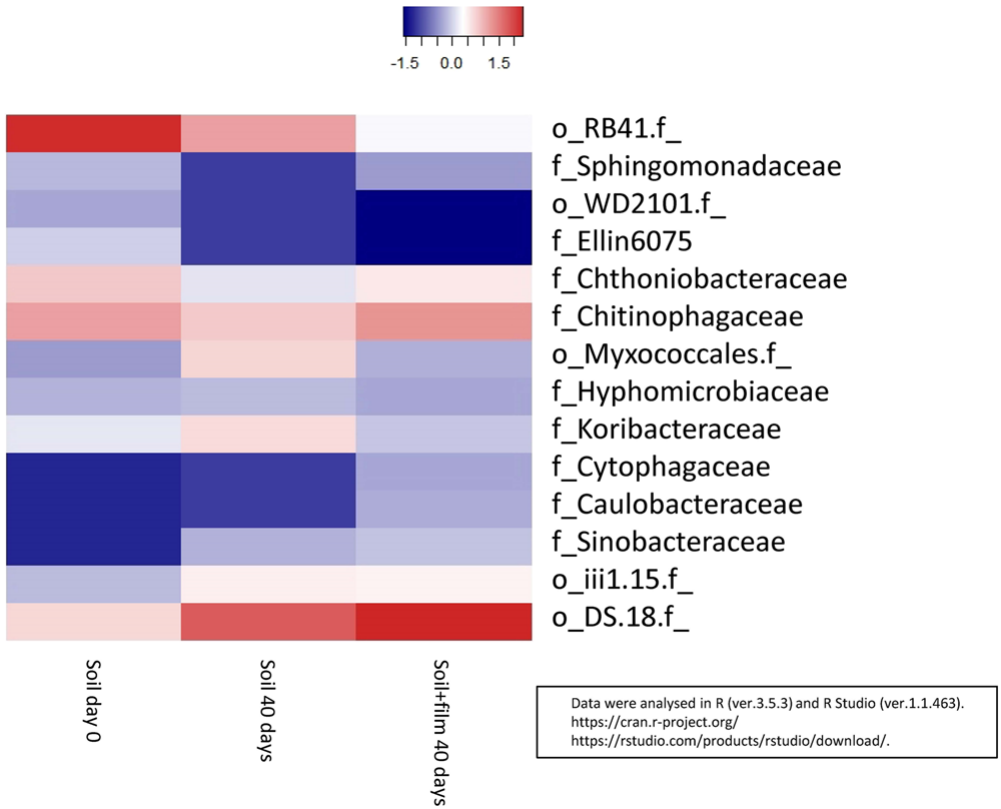
Changes in the microbial community in film burial in soil (family level).

We analysed the microbial community in the initial soil, the soil itself, which was left for 40 days (with humidification) and the soil with woody film added, which was left for 40 days (with humidification). The results are shown in Supplementary Table S2 and Fig. 3. *Acidobacteria* and *Proteobacteria* were dominant in the initial soil, and there was almost no change when we left the soil alone for 40 days. Furthermore, there was no significant change in the microbial community when the film was buried in the soil for 40 days. Initially, we expected that the abundance of bacteria contributing to biodegradation might be greatly increased, but since there was no major change in the microbial community, we cannot suggest which bacteria mainly contributed to the film degradation based on these results.

However, previous research has shown that *Acidobacteria* can degrade hemicellulose, and *Proteobacteria* can degrade hemicellulose and lignin^9^. Moreover, *Alphaproteobacteria*, *Acidobacteria* and *Actinobacteria* were dominant in dead wood^22,23^. Therefore, we concluded that *Acidobacteria* and *Proteobacteria*, which were often found in soil in this study, might contribute to the degradation of woody film.

In addition, almost no change in the microbial community was found in the heat map (Fig. 3). The bacteria with increased relative abundance after 40 days (such as *Acidobacteria*, class_ *iii1.8*, order_ *DS.18*) increased regardless of the presence or absence of the film. Therefore the bacteria are unlikely to play a major role in film degradation. *Rhizobiales* (*Alphaproteobacteria*), which became more abundant with the progress of wood decay in dead wood^22^, were present in the soil (family *Hyphomicrobiaceae* in Fig. 3), but they did not become more abundant during the experimental period, and it remains unclear whether or not *Rhizobiales* were involved in degradation of the woody film.

### Carbon dioxide (CO_2_) and methane (CH_4_) generation during film biodegradation

**Figure 4:**
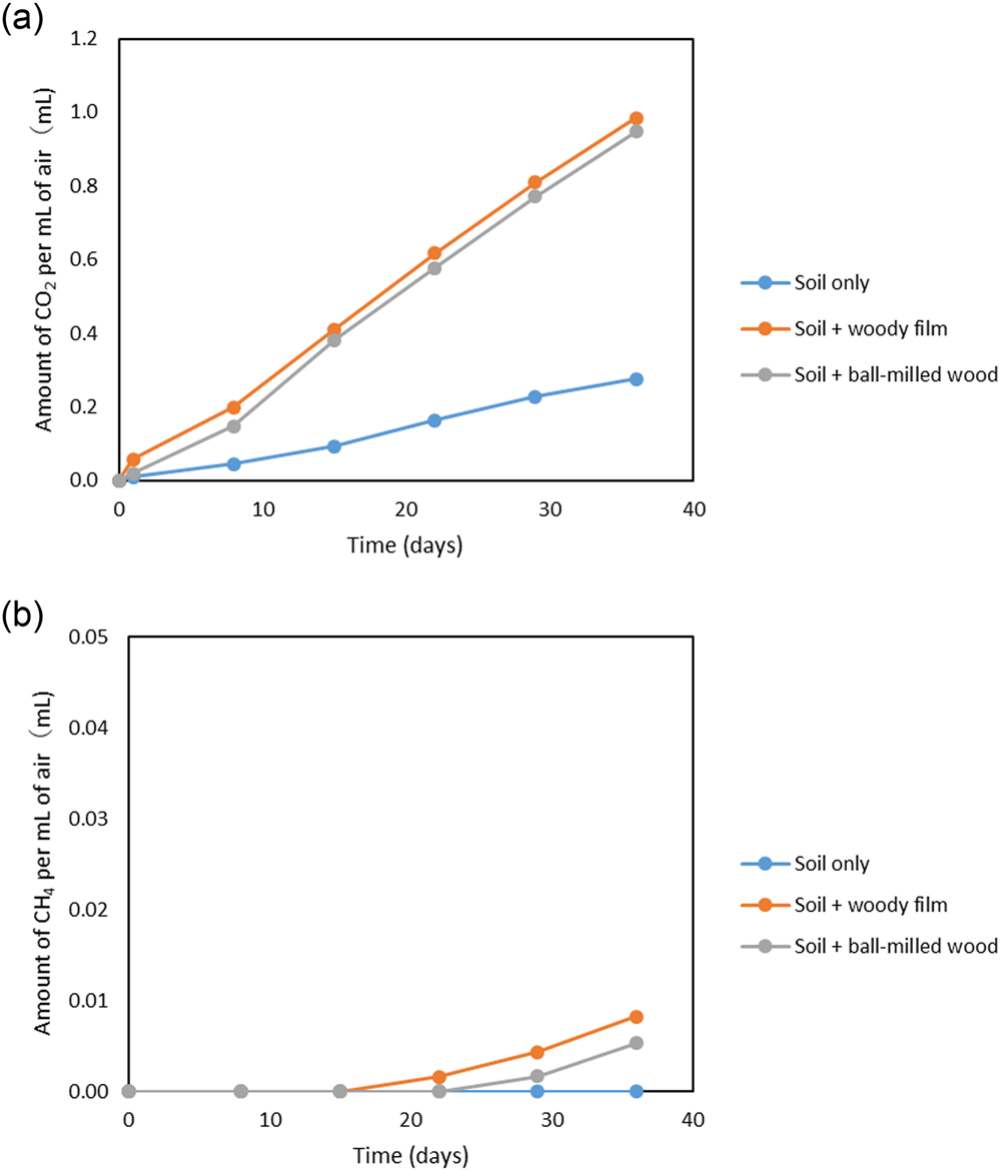
(**a**) Amount of carbon dioxide (CO_2_) and (**b**) methane (CH_4_) generated (integration of weekly measurements) during soil incubations.

To confirm that the material was not only brittle but apparently biodegraded and disintegrated in the soil, we performed a quantitative measurement of the CO_2_ and CH_4_ generated during film biodegradation. We confirmed that CO_2_ was generated even in the soil-only control test, but CO_2_ considerably increased compared with the soil-only treatment when both the film and the ball-milled Japanese Beech wood were added to the soil (Fig. 4(a)).

When soil microorganisms decompose the organic matter in the soil and use it as an energy source, CO_2_ is generated (soil respiration). The more easily degradable the organic matter, the more CO_2_ is generated (the respiration activity is high)^24^. Therefore, this film was considered to be as easily decomposed as the ball-milled Japanese Beech raw material. We presume that CO_2_ is generated not only by soil respiration but also partly by oxidation of the carbon component of the biodegradable film to CO_2_. In addition, when film or ball-milled wood was added to the soil, small amounts of CH_4_ were generated after 3 weeks (Fig. 4(b)). The CH_4_ generated was considered to be related to the transformation in film or wood carbon components. The film was biodegraded at the end of the study (after 5 weeks incubation) and apparently collapsed and disappeared (Supplementary Fig. S1).

During this measurement, approximately 20% CO_2_ (concentration in the air) was generated in one week in the sample containing woody film and ball-milled wood, and from this we inferred a concomitant decrease in oxygen (O_2_). Therefore, air was replaced weekly to compensate for the decreased O_2_ in this test as described in the methods section.

**Figure 5:**
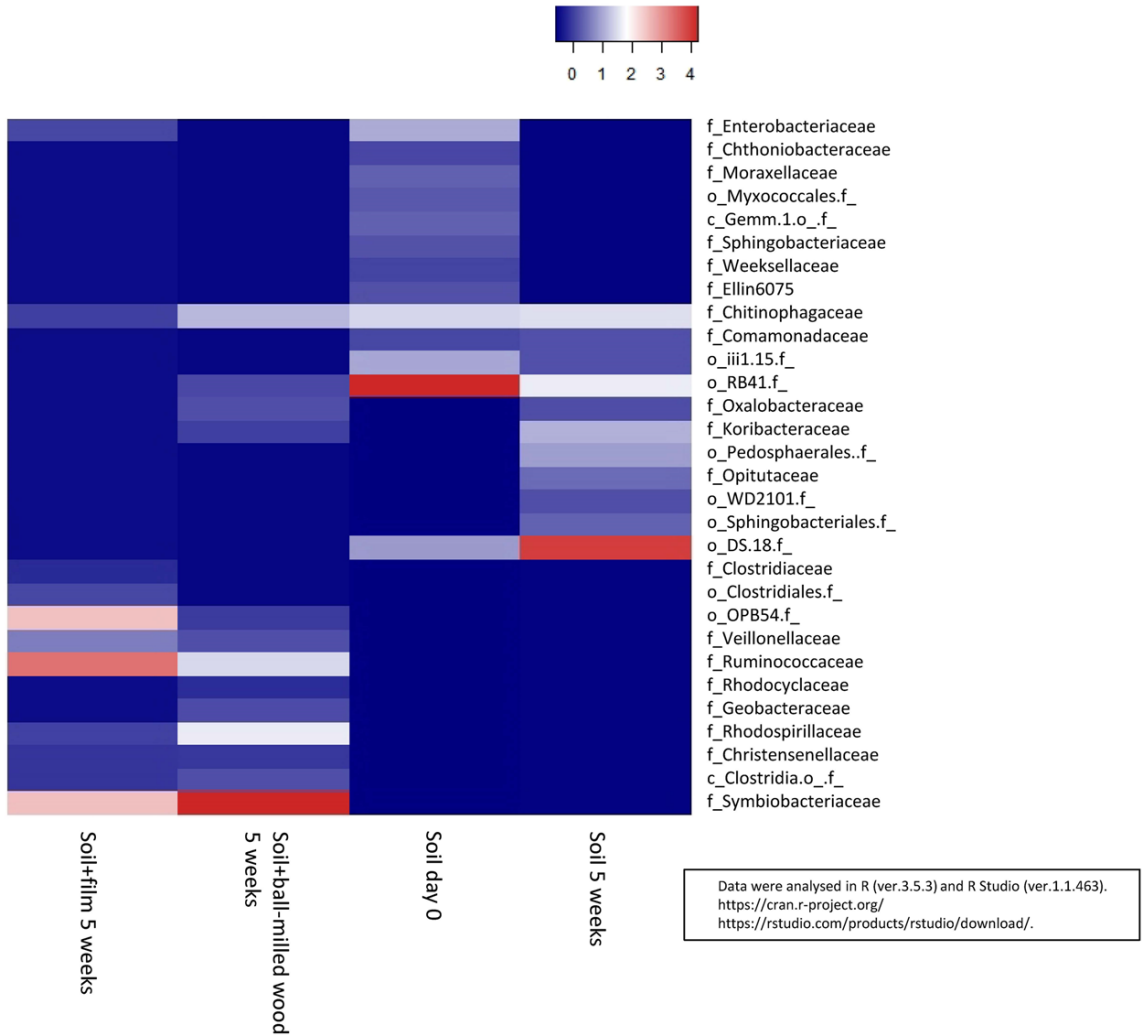
Changes in the microbial community during quantitative carbon dioxide measurement (family level).

Changes in the microbial community before and after the soil incubation test are shown in Supplementary Table S3. In Supplementary Table S3, we selected only one sample from the soil-alone and soil with film samples from the duplicate CO_2_ measurement test for the microbial community analysis. This is because the two samples showed almost the same behavior in the CO_2_ measurement test, and it would thus be sufficient to analyse microbial community on only one sample for each. Before the test, *Proteobacteria*, *Acidobacteria* and *Bacteroidetes* were dominant in the soil (soil day 0). The soil-only sample showed almost no change from before the test, but the film and ball-milled wood samples showed a marked decrease in relative abundance of *Acidobacteria*, *Actinobacteria* and *Verrucomicrobia*, and a marked increase in the relative abundance of *Firmicutes*. The microbial community comparison on the heat map clearly shows that anaerobic bacteria became dominant (Fig. 5). *Clostridia* (*Clostridiales*) among the *Firmicutes* became dominant, especially families *Ruminococcaceae* and *Symbiobacteriaceae*. *Clostridia* are known to be anaerobic, which suggests that the samples became anaerobic.

We observed the appearance of the soil every week. After two weeks, the colour of the soil-only sample was reddish brown (10YR 4/4; “Standard Soil Color Charts”^25^), whereas the samples with the film and ball-milled wood turned greyish brown (10YR 4/1) (Supplementary Fig. S2).

We suggest that the soil became anaerobic despite the replacement of the air every week for replenishment of O_2_, because the replenishment was insufficient and also the soil was not stirred. Anaerobic conditions might cause growth of anaerobic bacteria, change of soil colour, and generation of CH_4_.

However, it is interesting that the woody film biodegraded at 37 °C after 5 weeks even under these anaerobic conditions (Supplementary Fig. S1). Since *Ruminococcaceae* are known to be present in the stomach of herbivores and are capable of degrading cellulose under anaerobic conditions, we presumed that they degraded the film.

However, it should be noted that not only anaerobic bacteria but also aerobic bacteria contributed to biodegradation because air was replaced every week and CO_2_ was generated.

### The potential of woody film as a sustainable material

This study found that a film with almost the same composition as wood did not degrade in cultured hand bacteria but did degrade in cultured soil bacteria, and both in the case of simple burial in soil and burial in soil with measurement of CO_2_ under anaerobic conditions.

The woody film decomposed easily in the soil of various conditions in this study. In addition, we confirmed that the soil in which the film was buried generated a large amount of CO_2_ when compared with the soil alone. This CO_2_ generation suggests that the film is a readily degradable organic substance.

The above results indicate that the film does not disintegrate at the time of use, but is easily decomposed when buried in the soil after use. Therefore we expect that the film can be used as a sustainable raw material and can be used such as packaging containers, agricultural supplies, and even works of art, which could greatly change everyday life in the future.

## Methods

### Method to make woody film

Japanese Beech wood was ball-milled using a ball-mill (MB-1, manufactured by Chuo Kakoki Co., Ltd., Aichi, Japan) while cooling with water for 48 h under a nitrogen gas atmosphere. Ball-milled wood (0.2 g) and formic acid (3.8 g) were mixed and then stirred at room temperature for approximately 5 days. The resulting 5% by weight solution was dried at room temperature on a polyethylene terephthalate (PET; TORAY Tetoron film, Tokyo, Japan) substrate to obtain a film^5^.

### Cultivation of soil bacteria and hand bacteria

#### Soil bacteria

Approximately 5 mL of soil taken from Kanazawa University was put in a 50 mL tube, and adequate ultrapure water was added and subjected to ultrasound. It was left for about 5 min, and approximately 10 mL of the supernatant was mixed with 500 mL of culture medium, the composition of which is presented in Supplementary Table S4.

#### Hand bacteria

With the cooperation of six people (one man and five women) in their 20 s to 40 s, bacteria were scraped off the surface of the hand surface using a cotton swab for approximately 2 min with 1 L of liquid culture medium (composition was in Supplementary Table S4). Finally, the people rubbed both of their hands in liquid culture medium.

Both samples were stirred at 38 °C and the turbidity was measured with a spectrophotometer (DR 3900, HACH, Colorado, USA) after 1, 2 and 3 days. We confirmed that the turbidity increased after 1 day when compared with that at day 0, but after 2–3 days, the turbidity was almost the same as after 1 day and the bacterial growth had reached a steady state. In addition, microbial community analysis of the solution after growth was performed. Details of the microbial community analysis are provided below.

After both the soil and hand-grown bacteria were filtered, the culture medium was removed by washing with phosphate buffered salts, and liquid medium (composition given in Supplementary Table S4) without organic medium was added again. A woody film cut to 25 mm × 25 mm (approximately 0.05 g) was soaked in the liquid culture medium. The film was attached to the top of the beaker with a thread to ensure that the film touched the liquid on all surfaces and did not stick to the bottom.

The samples were placed in a thermostat at 37 °C and continuously stirred. We observed the appearance of the film every week.

### Woody film buried in the soil

The soil was taken from the site of Kanazawa University, and the original moisture content of the soil was determined by drying at 110 °C for 24 h followed by quantifying the difference between the wet and dry weights. The water originally contained in soil was approximately 7.3%. The water content of the soil was amended to approximately 18.3% through further addition of water. The moisture content of the soil was calculated as follows: weight of water/(weight of soil + weight of water).

**Figure 6:**
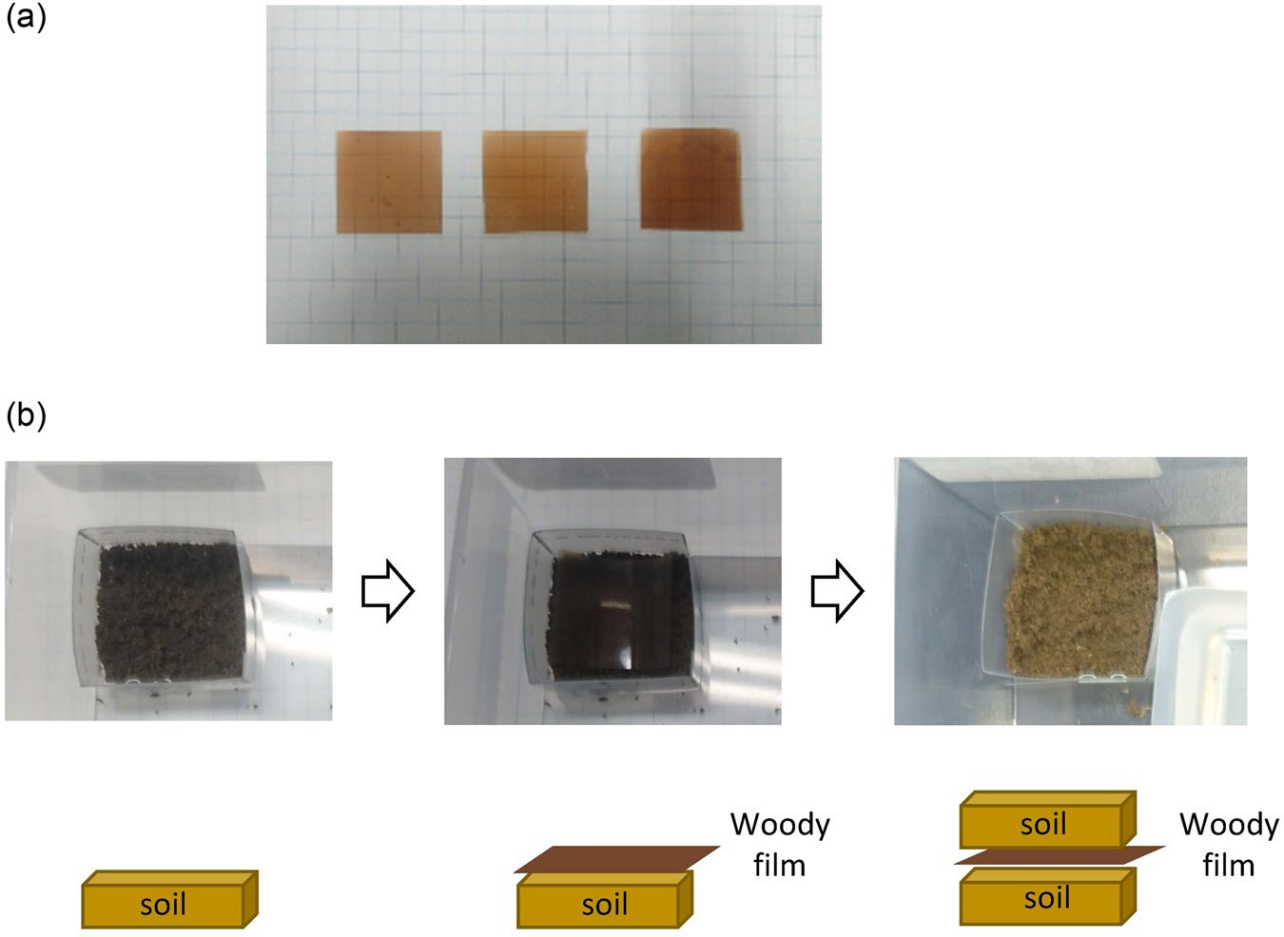
(**a**) Woody films to be buried in the soil and (**b**) stages used to bury the film in the soil.

The woody film was cut to 25 mm × 25 mm (Fig. 6(a)) and the weights of pieces were 0.05–0.08 g. To prevent soil dispersion, a plastic plate was used to form a mould, which surrounded the soil. Approximately 2 g of soil was placed in the mould, then a piece of woody film was placed on the soil surface, then another approximately 2 g of soil was placed on top of the film to sandwich it (Fig. 6(b)). The mould was placed in a plastic container with a lid, which contained a water dish to prevent the soil drying at room temperature. Additional water was sprayed onto the mould surface once every 1 or 2 weeks. The three samples (one sample was wet and two samples were dry) and a soil-only sample as a reference, were placed under the same conditions. We sprayed additional water onto all of the samples, but the drying rate was different depending on the sample and two samples became dry as a result. The container was large to ensure sufficient air volume. The microbial communities in the soil were analysed before the start of the test and again after 40 days. After 40 days, we observed whether or not the film had been degraded.

### CO_2_ measurement when the woody film was buried in the soil

Similar to the previous experiment, soil was taken from the site of Kanazawa University, and water was added after measuring the original moisture content (approximately 11.2%). As a result, the water content of soil was set to approximately 29.0%. Approximately 10 g of soil was placed in a 50-mL vial and the film was buried in the soil. The film was cut into 10 mm × 20 mm pieces. Five sheets of film per sample were added to the soil and the film weight per sample was approximately 0.1 g (Supplementary Fig. S3(a)).

The following samples were prepared: two samples containing the film, two samples using only soil as a reference, and one sample containing about 0.1 g of ball-milled wood powder from which the woody film was made (Supplementary Fig. S3(b)). The vial was capped with a rubber lid and an aluminium cap, and 20 mL of air was injected to pressurise. The pressurisation was performed to avoid difficulty in gas sampling and external air contamination when the pressure in the vial became negative. The samples were stored at 37 °C in an incubator, and after one day, the gas in the vial was measured by gas chromatography (GC). Details of the GC measurement are provided below. The gas in the vial was measured once a week thereafter. Since the amount of CO_2_ generated was large and the amount of O_2_ tended to decrease, we opened the lid of the vial to replace the air once a week. At the time of air replacement, 20 mL of air was injected as at the start of the test. The microbial community of the soil was analysed before the start of the test and after 5 weeks. After 5 weeks, we also observed whether or not the film had been degraded.

### GC measurement

The gas was measured using a Shimadzu GC-8A with flame ionisation detector. Argon gas was used as the carrier gas, the column was a packed SHINCARBON ST 50/80 column, the injection temperature was 130 °C, and the column temperature was constant at 100 °C. Standard curves were prepared for CO_2_ and CH_4_ by changing the amount of standard gas. The amount of sample injected was 1 mL.

### Microbial community analysis

DNA of the samples was extracted by using a Powersoil DNA Isolation Kit (Mo bio, CA, USA). The DNA samples were stored at −20 °C until analysis. First, polymerase chain reaction (PCR) analysis was carried out using universal primers, uni515F and uni806R^26^. The composition of the PCR mixture (25 μL) was as follows: 0.625 units of PrimeSTAR GXL DNA Polymerase (Takara, Kusatsu, Japan), 200 nM of dNTP mixture, 5 μL of PrimeSTAR GXL buffer, 0.5 μL of each primer and 1 ng/μL of DNA. The thermal cycle conditions were 25 cycles at 98 °C for 10 s, 55 °C for 15 s, and 68 °C for 45 s. The PCR amplicons were then purified by using Agencourt AMpure XP (Beckman Coulter, Pasadena, USA). The second PCR was conducted using Nextera XT DNA Library prep Kit (Illumina) following the reference guide. The amplicons were then purified and pooled for sequencing. Sequencing was performed using the Illumina MiSeq platform at Kanazawa University. The raw sequence data were analysed following Hewawasam *et al*.^27^. Data were clustered to each operational taxonomic unit (OTU) at the 97% similarity level, and the assignment of OTUs was carried out using Greengenes^28^ and PyNAST^29^.

### Creation of heat maps

Data were analysed in R^30^ and R Studio (R-Tools Technology, Ontario, Canada). The R and R Studio can be downloaded from the following URL: https://cran.r-project.org/, https://rstudio.com/products/rstudio/download/. We used the version 3.5.3 for R, and 1.1.463 for R Studio.

The OTUs with a low frequency of appearance were excluded, and the samples with a small number of total reads were excluded. Subsequently, the “rarefy” function was performed with the package “vegan”^31^ in order to compare samples with different numbers of reads. Heat maps are used for visualisation in data analysis and can be created from R’s “heatmap” function. In this study, the heat map was generated using the package “heatmap3”^32^, which allows customisation of heat maps.

## Supplementary information

Supplementary Information.

## References

[1] Vroman I, Tighzert L (2009). Biodegradable polymers. Materials (Basel)..

[2] Miki T, Takeuchi K, Sugimoto H, Kanayama K (2007). Performance Study of compact wood powder material processing for improved impact characteristics aiming at substitute for plastics. J. Mater. Process. Technol..

[3] Yamashita O, Yokochi H, Miki T, Kanayama K (2009). The pliability of wood and its application to molding. J. Mater. Process. Technol..

[4] Nishiwaki-Akine Y, Watanabe T (2014). Dissolution of wood in [small alpha]-keto acid and aldehydic carboxylic acids and fractionation at room temperature. Green Chem..

[5] Nishiwaki-Akine Y (2017). Transparent Woody Film Made by Dissolution of Finely Divided Japanese Beech in Formic Acid at Room Temperature. ACS Sustain. Chem. Eng..

[6] Sánchez C (2009). Lignocellulosic residues: Biodegradation and bioconversion by fungi. Biotechnol. Adv..

[7] Singh Adya P., Kim Yoon Soo, Singh Tripti (2016). Bacterial Degradation of Wood. Secondary Xylem Biology.

[8] Annele H (1994). Lignin-modifying enzymes from selected white-rot fungi: production and role from in lignin degradation. FEMS Microbiol. Rev..

[9] Kameshwar AKS, Qin W (2016). Recent developments in using advanced sequencing technologies for the genomic studies of lignin and cellulose degrading microorganisms. Int. J. Biol. Sci..

[10] Koeck DE, Pechtl A, Zverlov VV, Schwarz WH (2014). Genomics of cellulolytic bacteria. Curr. Opin. Biotechnol..

[11] Brumm PJ (2013). Bacterial genomes: What they teach us about cellulose degradation. Biofuels.

[12] La Reau AJ, Suen G (2018). The Ruminococci: key symbionts of the gut ecosystem. J. Microbiol..

[13] Zimmermann W (1990). Degradation of lignin by bacteria. J. Biotechnol..

[14] Li J, Yuan H, Yang J (2009). Bacteria and lignin degradation. Front. Biol. China.

[15] Huang XF (2013). Isolation and characterization of lignin-degrading bacteria from rainforest soils. Biotechnol. Bioeng..

[16] Xu R (2018). Lignin depolymerization and utilization by bacteria. Bioresour. Technol..

[17] Zhang XZ, Zhang YHP (2010). One-step production of biocommodities from lignocellulosic biomass by recombinant cellulolytic Bacillus subtilis: Opportunities and challenges. Eng. Life Sci..

[18] Fierer N, Hamady M, Lauber CL, Knight R (2008). The influence of sex, handedness, and washing on the diversity of hand surface bacteria. PNAS.

[19] Cho I, Blaser MJ (2012). The human microbiome: At the interface of health and disease. Nat. Rev. Genet..

[20] Elizabeth A G (2006). Topographical and Temporal Diversity of the Human Skin Microbiome. Science.

[21] Grice EA, Segre JA (2011). The skin microbiome. Nat. Rev. Microbiol..

[22] Hoppe B (2015). A pyrosequencing insight into sprawling bacterial diversity and community dynamics in decaying deadwood logs of Fagus sylvatica and Picea abies. Sci. Rep..

[23] Valášková V, De Boer W, Klein Gunnewiek PJA, Pospíek M, Baldrian P (2009). Phylogenetic composition and properties of bacteria coexisting with the fungus Hypholoma fasciculare in decaying wood. ISME J..

[24] Kopeć M, Gondek K, Baran A (2013). Assessment of respiration activity and ecotoxicity of composts containing biopolymers. Ecotoxicol. Environ. Saf..

[25] Sugita R, Marumo Y (1996). Validity of color examination for forensic soil identification. Forensic Sci. Int..

[26] Caporaso JG (2012). Ultra-high-throughput microbial community analysis on the Illumina HiSeq and MiSeq platforms. ISME J..

[27] Hewawasam C, Matsuura N, Maharjan N, Hatamoto M, Yamaguchi T (2017). Oxygen transfer dynamics and nitrification in a novel rotational sponge reactor. Biochem. Eng. J..

[28] DeSantis TZ (2006). Greengenes, a chimera-checked 16S rRNA gene database and workbench compatible with ARB. Appl. Environ. Microbiol..

[29] Caporaso JG (2010). PyNAST: A flexible tool for aligning sequences to a template alignment. Bioinformatics.

[30] Beckerman, A P.; Childs, Dylan Z.; Petchey, O. L. Getting started with R: an introduction for biologists. second edition. (Oxford University Press., 2017).

[31] Dixon P (2003). Computer program review VEGAN, a package of R functions for community ecology. J. Veg. Sci..

[32] Zhao, S., Guo, Y., Sheng, Q. & Shyr, Y. Advanced Heat Map and Clustering Analysis Using Heatmap3. *Biomed. Res. Int*. **2014**, (2014). 10.1155/2014/986048 PMC4124803 25143956

