# Supplementary material for: Biodegradability of woody film produced by solvent volatilisation of Japanese Beech solution
Source: Sci Rep. 2020 Jan 16;10:476. doi: 10.1038/s41598-019-57228-7 (PMC6965195; doi:10.1038/s41598-019-57228-7)
Supplement: Supplementary file 1 — Supplementary Information. [file 41598_2019_57228_MOESM1_ESM.pdf]

Supporting Information

Biodegradability of woody film produced by solvent volatilisation of Japanese Beech solution

Yuri Nishiwaki-Akine[1*], Sui Kanazawa[2], Norihisa Matsuura[3], and Ryoko Yamamoto-Ikemoto[3]

[1]Career Design Laboratory for Gender Equality, [2]Division of Environmental Design, Graduate School of Natural Science and Technology, [3]Faculty of Geosciences and Civil Engineering, Institute of Science and Engineering, Kanazawa University, Kakuma-machi, Kanazawa 920-1192, Japan.
*nishiwaki@staff.kanazawa-u.ac.jp.

This supporting information contains 9 pages, 4 tables and 3 figures.

Supplementary Table S1. Main soil cultures and hand cultures (Family level)

	relative abundance	
	hand	soil
k__Bacteria		
p__Proteobacteria;c__Gammaproteobacteria;o__Enterobacteriales;f__Enterobacteriaceae	41.9%	0.0%
p__Proteobacteria;c__Gammaproteobacteria;o__Pseudomonadales;f__Pseudomonadaceae	14.8%	0.0%
p__Firmicutes;c__Bacilli;o__Bacillales;f__Planococcaceae	13.3%	0.0%
p__Firmicutes;c__Bacilli;o__Bacillales;f__Paenibacillaceae	12.8%	1.1%
p__Firmicutes;c__Clostridia;o__Clostridiales;f__[Tissierellaceae]	11.6%	0.0%
p__Firmicutes;c__Clostridia;o__Clostridiales;f__Clostridiaceae	4.3%	0.0%
p__Firmicutes;c__Clostridia;o__Clostridiales;f__Lachnospiraceae	0.9%	0.0%
p__Firmicutes;c__Bacilli;o__Bacillales;f__Bacillaceae	0.1%	70.1%
p__Firmicutes;c__Bacilli;o__Bacillales;f__Staphylococcaceae	0.0%	22.9%
p__Firmicutes;c__Bacilli;o__Lactobacillales;f__Enterococcaceae	-	4.3%
p__Actinobacteria;c__Actinobacteria;o__Actinomycetales;f__Micrococcaceae	-	1.3%
p__Firmicutes;c__Bacilli;o__Lactobacillales;f__Streptococcaceae	-	0.2%
p__Firmicutes;c__Clostridia;o__Clostridiales;f__Gracilibacteraceae	0.3%	-
Others	0.1%	0.0%
Total	100.0%	100.0%

Supplementary Table S2. Changes in the microbial community in film burial in soil (Phylum level)

	relative abundance		
	Soil day 0	Soil 40 days	Soil + Film 40 days
k__Bacteria			
p__Acidobacteria	28.7%	29.3%	22.7%
p__Proteobacteria	27.6%	28.1%	31.6%
p__Verrucomicrobia	8.0%	9.0%	11.1%
p__Bacteroidetes	7.5%	8.0%	9.3%
p__Planctomycetes	7.1%	7.2%	6.9%
p__Actinobacteria	6.5%	3.7%	5.5%
p__Chloroflexi	4.6%	3.4%	2.6%
p__Gemmatimonadetes	2.3%	2.1%	2.0%
p__TM7	1.4%	0.8%	1.3%
p__Armatimonadetes	1.4%	1.0%	1.2%
p__WS3	1.3%	1.5%	0.9%
p__Firmicutes	0.7%	0.1%	0.3%
p__Cyanobacteria	0.5%	0.9%	0.8%
p__Nitrospirae	0.5%	1.5%	0.8%
p__Chlorobi	0.3%	0.5%	0.2%
p__FCPU426	0.2%	0.8%	0.4%
p__Chlamydiae	0.1%	0.2%	0.2%
p__AD3	0.1%	0.0%	0.1%
p__Elusimicrobia	0.1%	0.4%	0.3%
p__Fibrobacteres	0.1%	0.3%	0.6%
p__TM6	0.1%	0.1%	0.5%
p__Spirochaetes		0.4%	0.2%

k__Archaea			
p__Crenarchaeota	0.4%	0.5%	0.1%
Others	0.5%	0.3%	0.5%
Total	100.0%	100.0%	100.0%

Supplementary Table S3. Changes in microbial community during CO_2 quantitative measurement (Phylum level)

	relative abundance			
	Soil<br>day 0	Soil<br>5 weeks	Soil+Film<br>5 weeks	Soil+Wood<br>powder<br>5 weeks
k__Bacteria				
p__Proteobacteria	28.9%	23.2%	12.8%	27.3%
p__Acidobacteria	28.7%	32.0%	3.7%	7.7%
p__Bacteroidetes	12.1%	9.7%	2.7%	7.4%
p__Actinobacteria	6.0%	2.1%	0.5%	1.3%
p__Verrucomicrobia	5.3%	10.7%	1.2%	3.4%
p__Planctomycetes	3.8%	4.4%	1.4%	2.2%
p__Gemmatimonadetes	3.8%	2.3%	0.7%	1.5%
p__Chloroflexi	2.8%	2.5%	0.6%	1.1%
p__Nitrospirae	1.5%	1.8%	0.2%	0.3%
p__Cyanobacteria	1.1%	0.7%	0.1%	0.4%
p__TM7	1.1%	0.6%	0.0%	0.1%
p__WS3	1.0%	1.9%	0.1%	0.4%
p__Armatimonadetes	0.9%	0.7%	0.2%	0.1%
p__Firmicutes	0.6%	3.0%	74.4%	46.1%
p__Chlorobi	0.3%	0.4%	0.03%	0.04%
p__BHI80-139	0.1%	0.0%	0.0%	0.0%
p__OP3	0.1%	0.3%	0.0%	0.0%
p__Elusimicrobia	0.03%	0.5%	0.02%	0.03%
p__Fibrobacteres	0.03%	0.4%	0.0%	0.01%
p__OD1	0.03%	0.3%	0.03%	0.07%
p__WS2	0.0%	0.2%	0.0%	0.01%
p__NKB19	0.0%	0.2%	0.0%	0.0%
p__OP11	0.0%	0.1%	0.0%	0.0%

k__Archaea				
p__Crenarchaeota	0.9%	0.9%	0.1%	0.2%
p__Euryarchaeota	0.4%	0.0%	0.9%	0.1%
p__[Parvarchaeota]	0.0%	0.2%	0.0%	0.0%
Others	0.5%	0.8%	0.2%	0.2%
Total	100.0%	100.0%	100.0%	100.0%

Supplementary Table S4. The composition of the culture medium

		g/L
Organic medium	Hipolypeptone	2.5
	Tryptone	2.5
	Yeast extract	2.5
	Beef extract	2.5
	Glucose	2.5
Inorganic medium	K_2HPO_4	0.04
	KH_2PO_4	0.04
	$NaHCO_3$	0.8
	NaCl	0.08
	$CaCl_2$	0.0075
	$MgSO_4 \cdot 7H_2O$	0.02
trace element	EDTA	0.00065
	$FeSO_4 \cdot 7H_2O$	0.0009
	$ZnSO_4 \cdot 7H_2O$	0.00043
	$CoCl_2 \cdot 6H_2O$	0.00024
	$MnCl_2 \cdot 4H_2O$	0.00099
	$CuSO_4 \cdot 5H_2O$	0.00025
	$NaMoO_4 \cdot 2H_2O$	0.00022
	$NiCl_2 \cdot 6H_2O$	0.00027
	H_3BO_3	0.000014
	$NaSeO_4 \cdot 10H_2O$	0.00021
Water	Water	residue

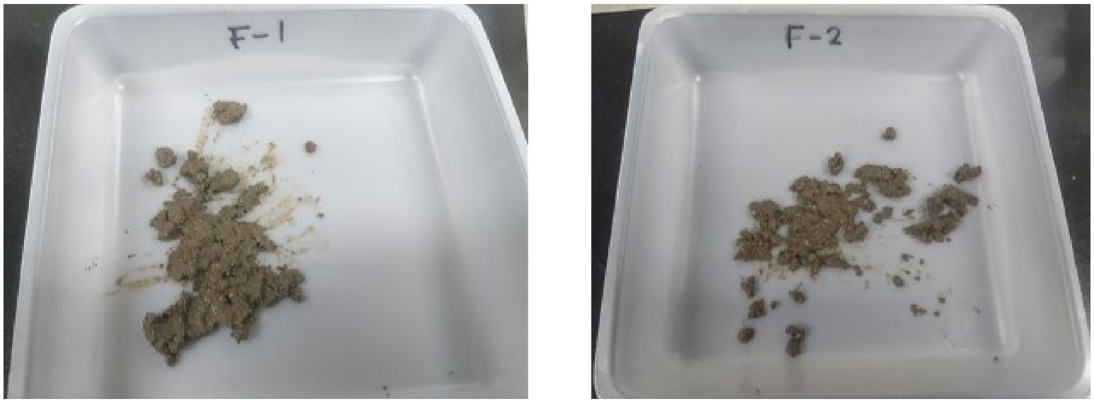

Supplementary Figure S1. The woody films were biodegraded in the soil after five weeks

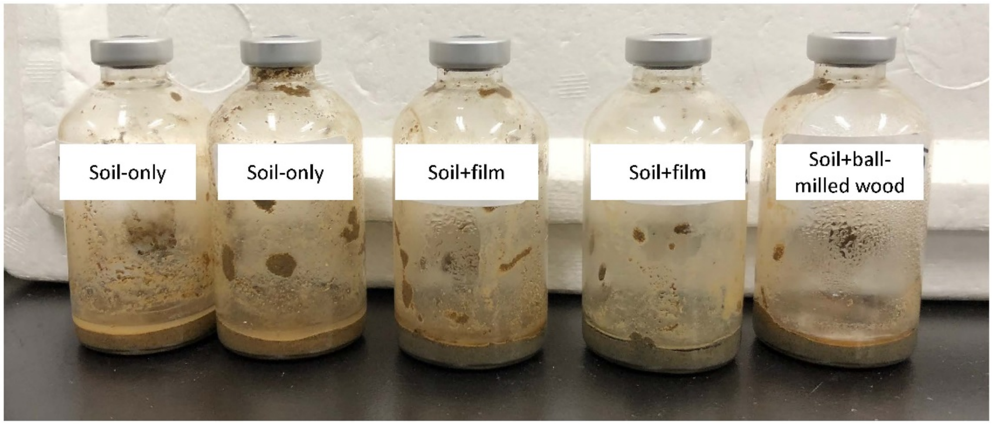

Supplementary Figure S2. The colour of soil after two weeks

(a)

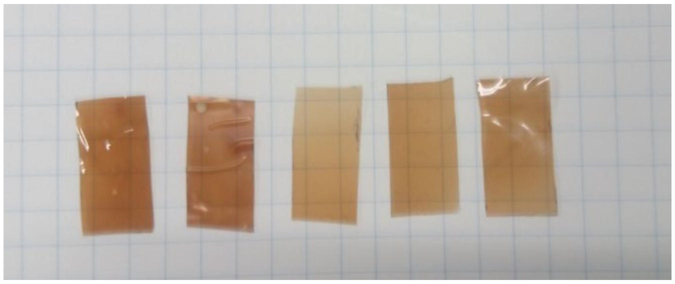

(b)

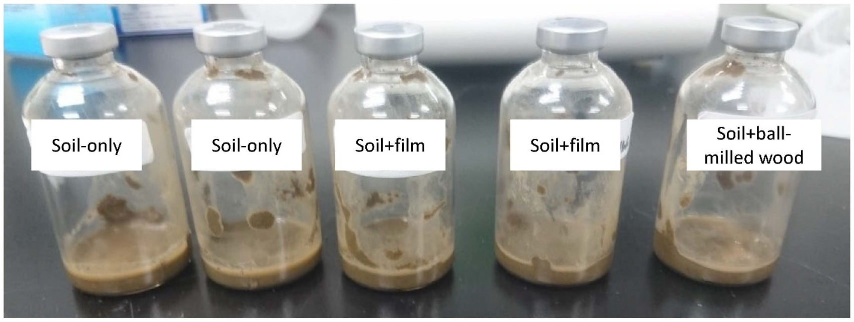

Supplementary Figure S3. (a) Woody films to be buried in the soil for carbon dioxide measurement and (b) the vials with rubber lids and aluminum caps
